# CFD–DEM method is used to study the multi-phase coupling slag discharge flow field of gas-lift reverse circulation in drilling shaft sinking

**DOI:** 10.1038/s41598-024-64519-1

**Published:** 2024-06-15

**Authors:** Longhui Guo, Hua Cheng, Zhishu Yao, Chuanxin Rong, Zongjin Wang, Xiaoyun Wang

**Affiliations:** 1 School of Civil Engineering and Architecture, Anhui University of Science and Technology, No.168 Taifeng Street, Shannan New District, Huainan, 232001 Anhui China; 2 School of Resources and Environmental Engineering, Anhui University, Hefei, 230022 China; 3 Anhui Provincial Key Laboratory of Building Structure and Underground Engineering, Anhui Jianzhu University, Hefei, 230601 China; 4 China Coal Special Drilling Co., Ltd., Hefei, 230001 China

**Keywords:** CFD–DEM, Multiphase coupling, Drilling shaft sinking, Gas lift reverse circulation, Slag discharge flow field, Civil engineering, Energy infrastructure

## Abstract

To elucidate the distribution law of the multiphase coupling slag discharge flow field in gas-lift reverse circulation during drilling shaft sinking, a numerical analysis model of gas–liquid–solid multiphase coupling slag discharge was established by CFD–DEM (Coupling of computational fluid dynamics and discrete element method) method, taking the drilling of North Wind well in Taohutu Coal Mine as an example. This model presented the distribution of the multiphase flow field in the slag discharge pipe and at the bottom hole, and was validated through experimentation and theoretical analysis. Finally, the impact of factors, including bit rotation speed, gas injection rate, air duct submergence ratio, and mud viscosity on the slag discharge flow field was clarified. The results indicated that the migration of rock slag at the bottom of the well was characterized by “slip, convergence, suspension, adsorption, and lifting”. The slag flow in the discharge pipe exhibited the states of “high density, low flow rate” and “low density, high flow rate”, respectively. The multiphase fluid flow patterns in the well bottom and slag discharge pipe were horizontal and axial flows, respectively. The model test of the gas lift reversed circulation slag discharge and the theoretical model of the bottom hole fluid velocity distribution confirmed the accuracy of the multiphase coupling slag discharge flow field distribution model. The rotation speed of the drill bit had the most significant impact on the bottom hole flow field. Increasing the rotation speed of the drill bit can significantly enhance the tangential velocity of the bottom hole fluid, increase the pressure difference between the bottom hole and annular mud column, and improve the adsorption capacity of the slag suction port. These findings can provide valuable insights for gas lift reverse circulation well washing in western drilling shaft sinking.

## Introduction

China possesses an abundant supply of coal resources, which are expected to serve as a “stabilizer” and “ballast stone” for the country’s long-term energy security^1,2^. As coal resources in the central and eastern regions gradually deplete and the western development strategy continues to advance, coal resource development has shifted towards western mining areas^3,4^. In recent years, large-scale construction of new coal mines has occurred in regions such as Xinjiang, Gansu, Inner Mongolia, and Shaanxi. These new mines are located in deep Cretaceous and Jurassic strata, which pose challenges for construction owing to their loose structure and abundant water. Therefore, special methods are required for shaft drilling^5,6^.

**Figure 1:**
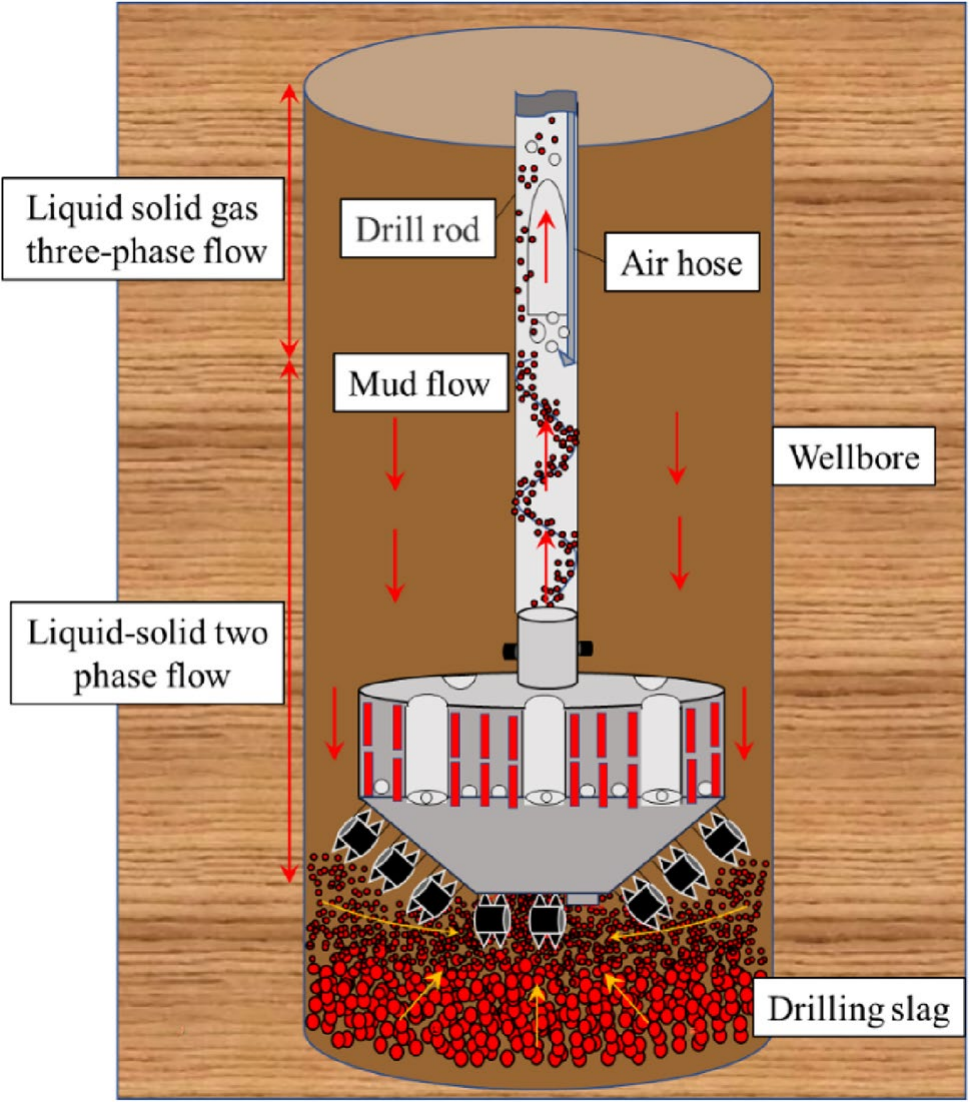
Schematic diagram of gas lift reverse circulation slag discharge.

Shaft sinking is a mechanized and automatic method for constructing mine shafts that pass through deep and unstable strata with a high-water content. The construction process involves mechanical rock breaking using a shaft drilling rig, circulating mud to wash and discharge slag, prefabricating the drilling shaft lining on the ground, suspending mud to sink the shaft lining, and filling and cementing behind the wall. The process of gas lift reverse circulation slag discharge is shown in Fig. 1. Compared with the freezing method, the drilling method offers advantages, such as high mechanization, a good working environment, and high wall quality, facilitating the well drilling without entering the well or compromising safety. This is a future direction for deep shaft sinking^7–9^. Shaanxi Yanchang Petroleum was the first to utilize the drilling method to construct the inlet and return vertical shafts of Cretaceous-Jurassic strata in Kekegai Coal Mine in the west, and has recently completed the construction of two wells^10,11^. However, several engineering challenges arise when drilling Jurassic mudstone, sandy mudstone, and other strata, including severe tool wear, poor slag discharge efficiency, and low drilling efficiency. Preliminary analysis suggests that there is inadequate understanding of the distribution of the liquid–solid–gas multiphase coupling slag discharge flow field. Additionally, the slag discharge speed in the gas lift reverse circulation cannot satisfy the requirements of efficient well completion. This deficiency is a significant factor that contributes to the low drilling and slag discharge efficiency. Therefore, it is imperative to urgently investigate and address this issue.

In recent years, experts and scholars in relevant fields, both domestically and internationally, have conducted numerous studies on the distribution law of drilling slag discharge flow fields and their influencing factors, achieving a series of findings. Liu et al.^12^, Feng et al.^13^, Zhang et al.^14^ and Fu et al.^15^ have investigated the mechanism of drilling slag and the optimization of drilling parameters, and have concluded that the existence of “three areas of drilling slag” is the fundamental reason for the difficulty of slag discharge. The factors affecting the slag discharge effect from high to low are the pressure of the slag discharge channel, aperture of the water inlet channel, length of the machine, pressure of the water inlet channel, and aperture of the slag discharge channel. Reasonable construction parameters for anchor hole drilling have been proposed. Huang et al.^16^ and Huang et al.^17^ have studied the distribution law of gas–solid two-phase flow field at the bottom of the hole during reverse circulation drilling with DTH hammer. Researchers believe that the drilling conditions at the bottom of the hole significantly affect the velocity, pressure, and turbulence characteristics of the gas-phase fluid in the flow field. When drilling through an intact formation, good reverse circulation can be maintained at the bottom of the hole. When drilling through a fractured formation, gas loss is severe, and the pumping effect deteriorates. Using air drilling technology, Qiu^18^ and Yao^19^ investigated the variation laws of wellbore pressure, velocity, and cutting density in the annulus of oil and gas wells under various bit types and drilling tool configurations. They analyzed the impacts of factors such as the gas injection rate, drill string rotation speed, and cutting particle size on the annulus flow field. Jiao et al.^20^, Chen^21^ and Meng^22^ conducted numerical simulations to study the distribution of air, foam, and fluid washing slag flow field in large diameter shaft boring machines and revealed the influence of various equipment parameters and drilling construction parameters on the fluid velocity and pressure at the bottom of the well.

Numerous studies have been conducted by scholars, both domestically and internationally, on the distribution law of drilling slag discharge flow fields. However, the majority of these studies have focused on small-sized drilled oil and gas wells, coal seam gas extraction holes, and roadway floor anchor holes. Furthermore, the slag discharge mode employed in these studies has primarily been pump suction reverse circulation or annular positive circulation slag discharge, and the slag discharge flow pattern has been observed to be either liquid–solid or gas–solid two-phase flow. The specifications for the drilling diameter, slag discharge method, slag discharge medium, and well washing parameters are distinct from those of large-bore coal mine vertical shaft drilling gas lift reverse circulation slag discharge operations, and the resulting data do not fulfill the necessary requirements.

In summary, this study focused on the research background of advanced drilling technology for the φ5.0 m shaft in the Taohutu Coal Mine in the western region, which utilized the CFD–DEM method (a coupling of computational fluid dynamics and discrete element method) to establish a multiphase numerical model for analyzing the gas–liquid–solid flow field and its main influencing factors in the vertical shaft drilling process of large diameter coal mines.

## Engineering background

Taohutu Coal Mine is situated in the eastern region of the Ordos Plateau in China, encompassing the middle and eastern sections of the Maowusu Desert. The general trend of the area is characterized by high elevations in the northwest and low elevations in the southeast. The planned annual production capacity of the mine is 8 Mt/a, and the coal seam occurrence stratum is a typical water-rich and weakly cemented rock stratum in the western portion of the area. The north wind shaft, currently under construction, is a drilling method shaft and represents the second such structure of its kind in the western region of China, following the central intake and return air shaft of the Kekegai Coal Mine. This mine also holds the distinction of having the deepest design depth achieved through drilling methods worldwide.

**Figure 2:**
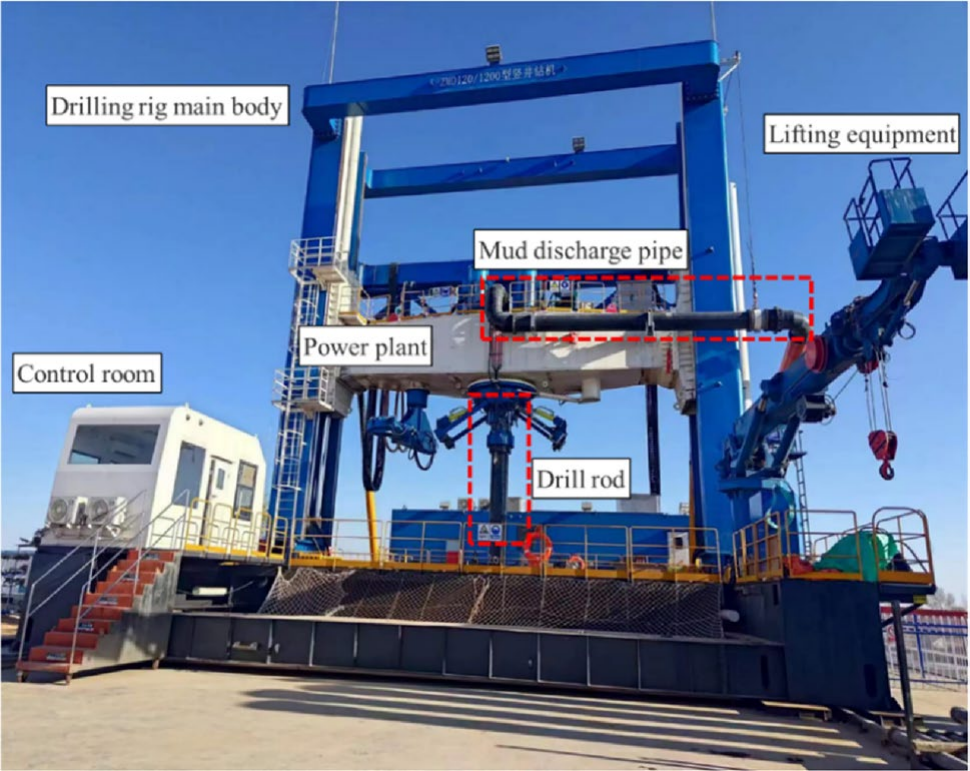
ZMD120/1200 shaft drilling rig.

**Figure 3:**
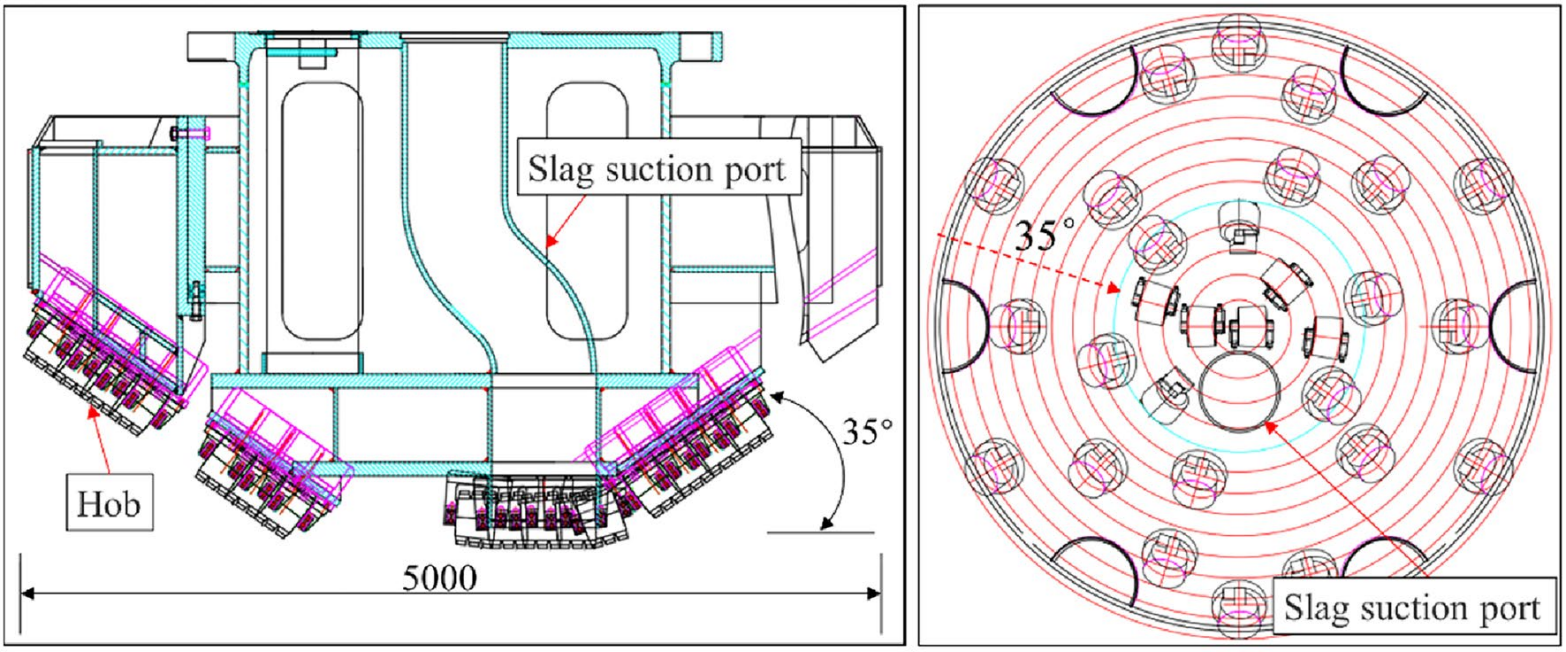
Layout of hob and slag suction port of φ5.0 m advanced bit.

The north wind vertical shaft has a design depth of 751 m and a net diameter of 6.5 m. The shaft passes through Quaternary, Cretaceous, Jurassic, and Triassic formations. The drilling diameter of the shaft is 9.4 m. The newly developed ZMD120/1200 shaft drill is employed for the two-stage drilling construction of φ5.0 m advanced drilling and φ9.4 m reaming drilling. Advanced drilling applies a truncated cone hob bit with a diameter of 5 m and cutting taper of 35°. The diameter of the truncated cone was 1.88 m, and there were 29 hobs on the drill bit. The slag suction port is arranged with a sweeping single slag suction port with a diameter of 0.61 m and an eccentricity of 0.52 m. The well washing method adopted the gas lift reverse circulation slurry suspension slag discharge process. Figure 2 shows the ZMD120/1200 shaft drilling rig. Figure 3 shows the cutter arrangement and slag suction port arrangement of the drill bit.

## Numerical simulation model

The gas-lift reverse circulation slag removal process constitutes a three-phase coupled flow of fluid-phase mud, air, and discrete phase rock slag. To investigate the distribution law of the coupled flow field, a numerical model of gas-lift reverse circulation slag removal has been established using the CFD–DEM method^23^. The distribution and migration of the fluid and discrete phases are calculated using simulation software Fluent and EDEM, respectively, and both are coupled through a compiled UDF coupling interface.

### Governing equation of multiphase flow

#### Fluid phase and gas phase

The flow of mud and air in the slag discharge flow field is characterized as a gas–liquid two-phase flow, which is subject to the conservation of fluid mass and Newton’s second law. Therefore, the Euler multiphase flow model was selected in Fluent to simulate fluid phases. The gas–liquid two-phase continuity equation and momentum conservation equation were established in the Euler coordinate system^24,25^, as shown below:

1 $$\frac{\partial }{\partial t}(\alpha_{i} \rho_{i} ){ + }\nabla \cdot (\alpha_{i} \rho_{i} v_{i} ) = 0,$$

2 $$\frac{\partial }{\partial t}(\alpha_{i} \rho_{i} v_{i} ) + \nabla \cdot (\alpha_{i} \rho_{i} v_{i} v_{i} ) = - \alpha_{i} \nabla P + \nabla \cdot \tau_{i} + \alpha_{i} \rho_{i} g - F_{D} ,$$

where $i$ represents liquid phase or gas phase; $\alpha_{i}$ is volume fraction of $i$ phase; $\rho_{i}$ is density of $i$ phase, kg/m^3^; $v_{i}$ is velocity of $i$ phase, m/s; $\tau_{i}$ is the stress–strain tensor of $i$ phase; $P$ is the interphase shared pressure, MPa; $g$ is the acceleration of gravity, m/s^2^. $F_{D}$ is the interphase interaction force, N.

Considering that the flow of well flushing fluid mud exhibited rheological characteristics of a non-Newtonian fluid, the power-law fluid model was selected. The rheological equation for this model is as follows:

3 $$\tau { = }K\gamma^{n} ,$$

where $\tau$ is the shear stress, Pa; $K$ is the viscosity coefficient, Pa·s; $\gamma$ is shear rate s^−1^; $n$ is the fluidity index.

#### Discrete phase

**Figure 4:**
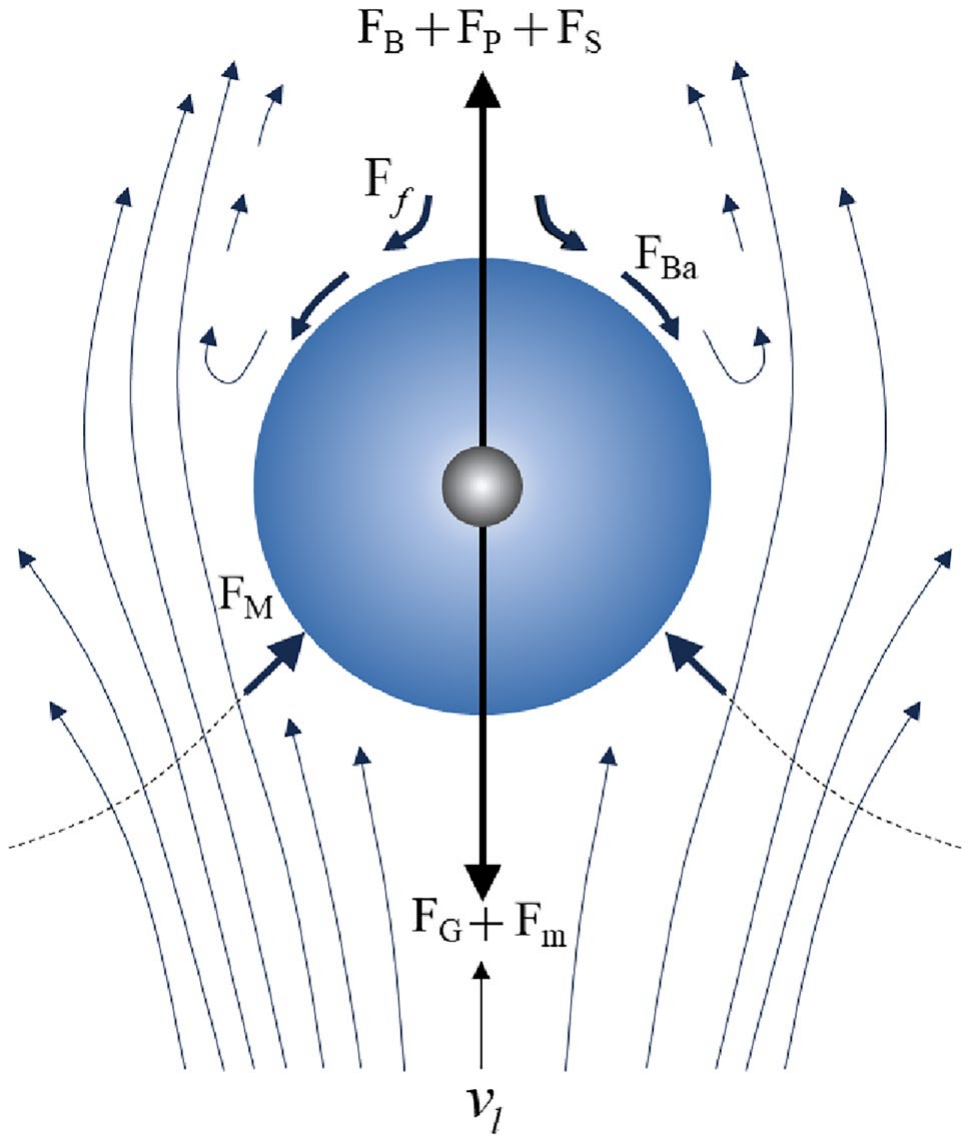
Stress diagram of rock slag.

The simulation software EDEM employing the discrete element method was utilized to analyze the distribution and flow of rock slag during the discharge process. At a rate of 3000 particles/s, rock slag was produced along the surface of the rock fracturing, with each slag particle corresponding to a discrete element. The movement of these particles is governed by Newton’s second law and the momentum conservation equation^26,27^. Figure 4 is the stress diagram of rock slag.

4 $$m\frac{{{\text{d}}v_{s} }}{{{\text{d}}t}} = F_{G} + F_{B} + F_{f} + F_{P} + F_{m} + F_{M} + F_{Ba} + F_{S} ,$$

where $v_{s}$ is the velocity of rock slag, m/s; $F_{G} ,F_{B} ,F_{f} ,F_{P} ,F_{m} ,F_{M} ,F_{Ba}$ and $F_{S}$ are the gravity, buoyancy, flow resistance, pressure gradient force, additional mass force, Magnus lift, Basset resistance, and Saffman lift exerted on the rock slag, respectively.

The conservation equation of the angular momentum of the rock slag is:

5 $$\frac{{\text{d}}}{{{\text{d}}t}}(Iw_{{\text{s}}} ) = T_{a} + T_{r} + T_{t} ,$$

where $I$ is the moment of inertia of rock slag, kg·m^2^; $w_{s}$ is angular velocity, rad/s; $T_{a}$, $T_{r}$ and $T_{t}$ are the axial, radial and tangential torques on the rock slag, respectively, N·m.

The Hertz–Mindlin non-slip contact model was applied in the EDEM to simulate the collision and extrusion between rock slag.

### Numerical model establishment

**Figure 5:**
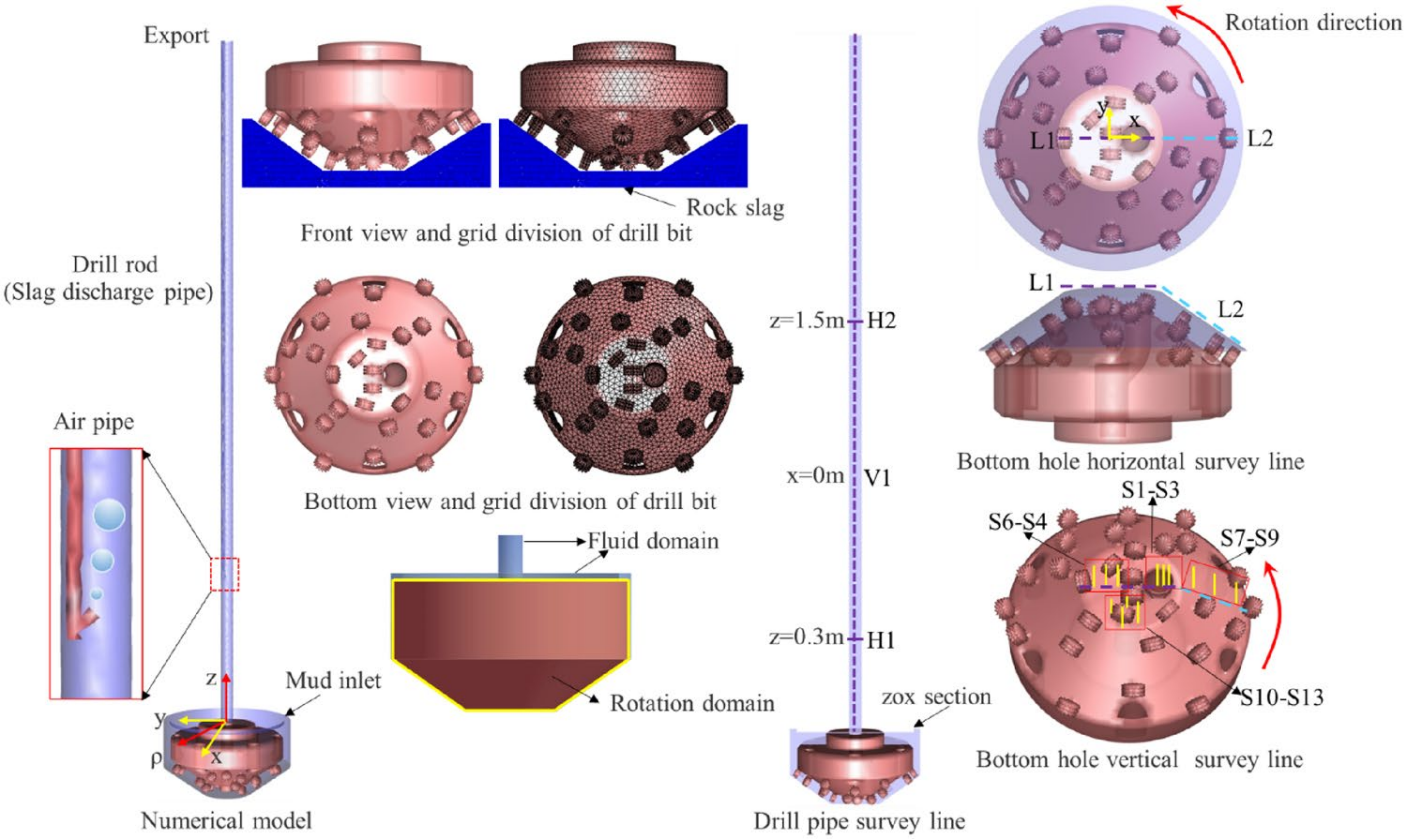
Numerical model of slag discharge and layout of survey lines.

Utilizing the construction technology of gas-lift reverse circulation slag removal via the drilling method and its corresponding structure, including the slag suction port and cutter arrangement of the φ5m advanced bit, a scaled numerical model of gas-lift reverse circulation liquid–solid–gas multiphase coupling slag removal was established for the Taohutu Coal Mine (scaled to 1/14 of the field model). This model primarily included the bottom hole, the drill bit, and the drill pipe. The drill bit was designated as the rotating domain and the wall of the drill pipe was designated as the rotating wall. Both rotate in the same direction and at the same speed, with the bottom hole and drill pipe interior serving as the fluid domain for mud to facilitate rock flow. A tetrahedral grid was employed to divide the numerical model, which was tested for grid quality and independence^28^. The Euler multiphase flow model and standard $k - \varepsilon$ turbulence model was utilized to calculate the slag discharge flow field, with uniform spherical particles incident along the rock breaking surface at the bottom of the well to simulate rock breaking and slag generation. Finally, the numerical simulation of the fluid and discrete phases was connected through the coupling interface, enabling the numerical simulation of the liquid–solid–gas multiphase coupling slag discharge. The modeling parameters were derived from the actual construction parameters and similar criteria, as illustrated in Table 1, and the numerical model of the slag discharge is depicted in Fig. 5.

**Table 1:** Model parameter selection.

Parameter	Symbols	Units	Prototype value	Model value
Bit rotation speed	$\omega$	r/min	5–10	30
Bit diameter	$D_{1}$	mm	5000	357
Bit height	$h$	mm	3000	214
Bit taper	$\alpha$	°	35	35
Mud density	$\rho_{l}$	kg/m^3^	1045–1307	1100
Mud viscosity	$\mu_{l}$	mPa·s	90–300	4
Rock slag density	$\rho_{s}$	kg/m^3^	2000–2600	2300
Rock slag diameter	$d_{s}$	mm	3–85	3
Rock slag Poisson’s ratio	$\nu$	–	–	0.3
Rock slag shear modulus	$G$	Pa	–	1 $\times$ 10^7^
Gas injection volume	$Q_{g}$	m^3^/h	3670–6480	7
Gas density	$\rho_{g}$	kg/m^3^	1.29	1.29
Bottom hole pressure	$P$	Pa	–	3 $\times$ 10^4^
Buried depth of air duct	$L_{1}$	mm	–	1700
Air duct diameter	$d_{1}$	mm	130	9.5
Drill pipe diameter	$d_{2}$	mm	560	40
Drill pipe length	$L_{2}$	mm	–	2150

## Distribution law of slag discharge flow field

**Figure 6:**
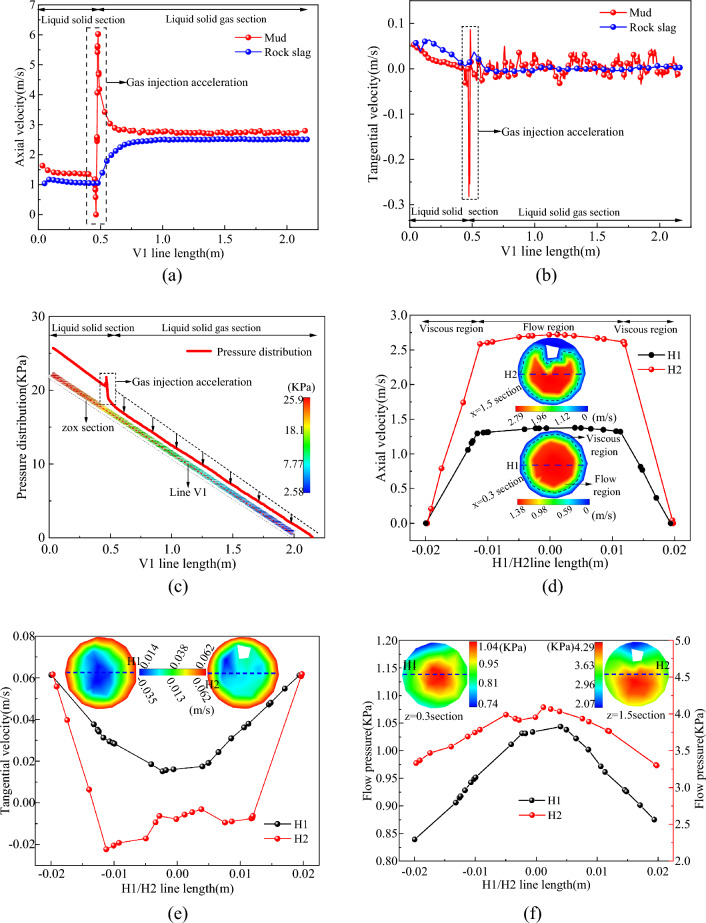
Distribution of flow field in slag discharge pipe. (**a**) Axial velocity on V1 survey line. (**b**) Tangential velocity on V1 survey line. (**c**) Pressure distribution on V1 survey line. (**d**) Axial velocity on H1/H2 survey line. (**e**) Tangential velocity on H1/H2 survey line. (**f**) Flow pressure on H1/H2 survey line.

The gas-lift reverse circulation slag discharge flow field during drilling sinking primarily involved flushing the circulating mud flow to the bottom of the well and transporting mud carrying rocks in the slag discharge pipe. To investigate the distribution pattern of the slag discharge flow field, survey lines were established at the bottom of the well and within the slag discharge pipe to monitor velocity and pressure distributions in the flow field. The survey line layout is illustrated in Fig. 6.

### Flow field in slag discharge pipe

Survey lines V1, H1, and H2 were strategically placed within the slag discharge pipe to monitor crucial information about the flow field within the pipe. Figure 6a–c present the velocity and pressure distribution data collected from survey line V1, whereas Fig. 6d–f depict the corresponding velocity and pressure distribution data from horizontal survey lines H1 and H2.

The analysis of Fig. 6a–c revealed that the fluid in the slag discharge pipe flowed in the form of liquid–solid and liquid–solid–gas. The flow form of the fluid underwent alterations when passing through the gas injection end, increasing the axial velocity of the mud and rock slag. The growth rates of these components were 95.6% and 148.4%, respectively. The density and pressure of the mixed fluid significantly decreased upon input of the gas phase (Fig. 6c). Under the impact of gas lift and pressure difference between the inside and outside of the slag discharge pipe, the mud carried rocks to achieve “gas injection acceleration” and obtained high flow rates and lift. The flow velocity of mud in the slag discharge pipe was consistently higher than that of rock slag, and the flow velocity of mud in the liquid–solid and liquid–solid–gas sections increased by 47.7% and 16.4%, respectively, compared to rock slag. Only under the condition that the flow velocity of the mud carrying the rock medium is sufficiently high and significantly greater than the sinking velocity of the rock slag in the mud can it be more advantageous for the rock slag to be discharged onto the ground^29^. Although the tangential velocity of the fluid in the slag discharge pipe was small, the turbulence degree of the mixed fluid intensified after the acceleration of gas injection, and the tangential velocity exhibited strong fluctuations.

Upon examining Fig. 6d–f, it becomes evident that the high-speed mixed fluid predominantly flowed in the mainstream region of the central part of the slag discharge pipe. In the viscous area near the pipe wall, the axial velocity of the fluid reduced rapidly due to viscous resistance, ultimately reaching zero. The tangential velocity distribution of the fluid was opposite to that of the axial velocity. Under the rotation of the slag discharge pipe, the tangential velocity of the inner wall surface was the highest, whereas that of the slag discharge pipe at the center was the lowest. In the slag discharge pipe section, the axial velocity of the fluid was greater than the tangential velocity. For instance, when comparing lines H1 and H2, the average axial velocity was 33 times and 96 times of the tangential velocity, respectively. This demonstrated that the fluid flow in the slag discharge pipe was predominantly axial. The flow pressure of the fluid reflects its kinetic energy variation. Along the section of the slag discharge pipe, the kinetic energy of the fluid peaked at the center and decreased linearly toward the periphery.

### Bottom hole flow field

#### Bottom hole horizontal flow field

**Figure 7:**
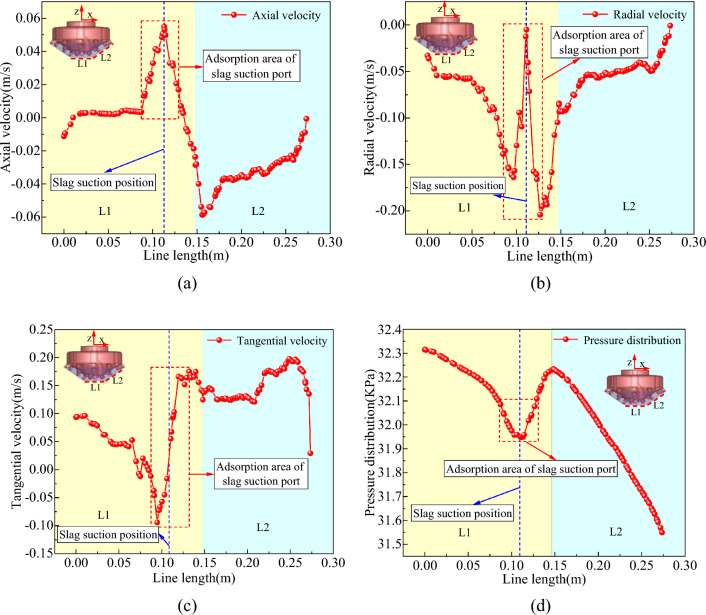
Distribution of horizontal flow field at bottom hole. (**a**) Axial velocity on L1/L2 survey line. (**b**) Radial velocity on L1/L2 survey line. (**c**) Tangential velocity on L1/L2 survey line. (**d**) Pressure distribution on L1/L2 survey line.

The horizontal survey lines L1 and L2 were positioned at the base of the well to gather data on the horizontal flow field at that location. Figure 7a–d illustrate the axial, radial, and tangential velocities and pressure distributions of the fluid on the horizontal survey lines at the bottom of the well.

From Fig. 7a–d, it can be inferred that the circulating mud flowed rapidly along the inclined rock-breaking surface at the bottom of the well owing to the combined effects of adsorption and gravity, and its axial and radial speeds continued to rise, thereby constantly replenishing mud in the adsorption area and achieving mud circulation and dynamic well cleaning. The upward axial flow of mud at the bottom of the well was mainly focused on the adsorption area near the slag suction port, whereas the radial flow of mud was the most pronounced on both sides of the slag suction port, with the velocity increasing as one approached the slag suction port. The tangential velocity of the mud at the center of the bottom hole was relatively small, whereas it increased with an increase in the rotation radius of the drill bit.

At the horizontal surface of the bottom hole, the primary mode of mud movement was radial flow, with velocities that were 7.3 times and 1.2 times greater than the axial and tangential velocities, respectively. At the inclined surface of the bottom hole, the primary mode of mud movement was tangential flow, with a velocity that was 4.5 times and 2.9 times greater than the axial and radial velocities, respectively. As a result, the primary mode of movement for the bottom hole fluid was horizontal flow.

The bottom hole pressure along the inclined plane increased from shallow to deep areas and decreased from the outer to the slag suction port along the radial direction at the horizontal plane of the bottom hole. The pressure in the adsorption area of the slag suction port was the lowest. The greater the pressure difference between the bottom hole horizontal plane and the annular mud column, the more easily the bottom hole mud and rock slag can be adsorbed and lifted.

#### Bottom hole vertical flow field

**Figure 8:**
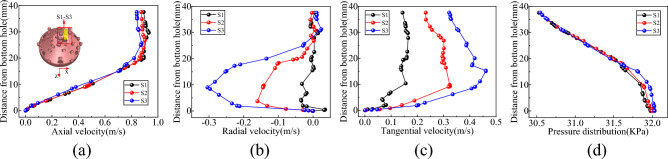
Velocity and pressure distribution on S1–S3 survey line at slag suction port. (**a**) Axial velocity. (**b**) Radial velocity. (**c**) Tangential velocity. (**d**) Pressure.

**Figure 9:**
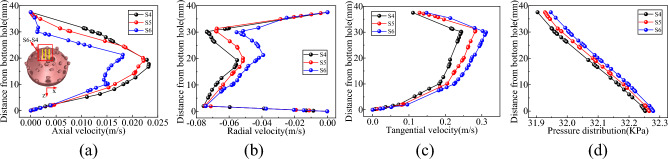
Velocity and pressure distribution on radial S4–S6 survey line of horizontal bottom hole. (**a**) Axial velocity. (**b**) Radial velocity. (**c**) Tangential velocity. (**d**) Pressure.

**Figure 10:**
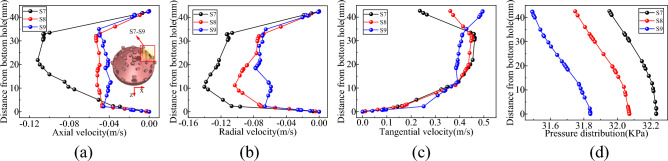
Velocity and pressure distribution on radial S7–S9 survey line of inclined bottom hole. (**a**) Axial velocity. (**b**) Radial velocity. (**c**) Tangential velocity. (**d**) Pressure.

**Figure 11:**
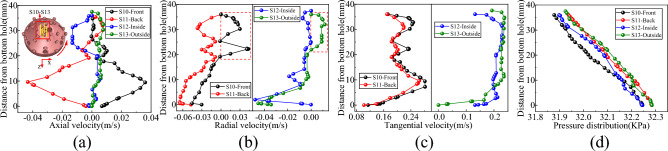
Velocity and pressure distribution on the survey line from S10 to S13 around the cutter. (**a**) Axial velocity. (**b**) Radial velocity. (**c**) Tangential velocity. (**d**) Pressure.

Vertical survey lines S1–S3, S4–S6, S7–S9, and S10–S13 were strategically positioned at the base of the well to monitor information about the flow field in various areas, including the slag suction port, radial direction of the horizontal bottom hole, radial direction of the inclined bottom hole, and periphery of the hob, as shown in Figs. 8, 9, 10 and 11.

As shown in Fig. 8, the primary motion of mud in the adsorption region of the slag suction port occurred in the axial direction. For instance, with reference to the S2 line, the average axial velocity was approximately 9.5 times and 2.5 times of the radial and tangential velocities, respectively. The axial velocity of the slurry at the center of the slag suction port was the most significant, decreasing gradually outward along the adsorption radius. Radial mud flow was nearly nonexistent at the center of the Slag suction port, while the radial and tangential velocities increased as the radius of adsorption expanded. Concurrently, the mud pressure within the adsorption zone increased in a linear fashion along the vertical axis of the well, exhibiting the least increase at the core of the slag suction port, where the dissimilarity in pressure between the mud and external annular mud column was at its zenith. In this region, the adsorption force of the slag suction port was the most potent, facilitating the absorption and elevation of mud and rock slag.

Based on the examination of Fig. 9, the vertical survey lines were oriented along the radial direction of the horizontal bottom hole. On the rock breaking surface and the surface of the drill bit, the axial and radial velocities of the mud were almost zero, whereas the axial velocity reached its maximum at the center of the bottom hole clearance. When the slag suction port was approached, the axial and radial velocities of the mud increased, and the tangential velocity of the mud increased with an increase in the rotary radius of the drill bit. At the same clearance depth at the bottom of the well, the well pressure decreased uniformly along the edge of the bit to the slag suction port and increased linearly in the vertical direction from the bit surface to the bottom of the well. Moreover, the well pressure growth rates at different radial positions were approximately equal.

It is evident from Fig. 10 that the circulating mud moved rapidly along the inclined rock-breaking surface towards the horizontal bottom hole, displaying an increase in both axial and radial speeds. Mud movement within the clearance range of the inclined bottom hole primarily consisted of tangential flow. Taking the S2 survey line as an example, the average tangential velocity was approximately 8 times and 4.9 times the axial velocity and radial velocity, respectively. At a distance of 35–43 mm from the bottom hole’s surface. The direction of the mud corresponded to that of the drill bit, and the velocity increased proportionally with the rotation radius of the drill bit. Within the range of 12–35 mm from the bottom hole, the drag effect of the drill bit on the mud was comparable to its viscous resistance, resulting in a constant speed for mud movement. At distances of ≤ 12 mm from the bottom of the well, the viscosity of the mud assumed a dominant role, and its tangential velocity decreased as the distance from the bottom of the well decreased, ultimately reaching zero^30^. Additionally, the mud pressure at the inclined bottom hole exhibits significant variation, displaying uniform increases along both the well depth direction and the inclined rock breaking surface.

**Figure 12:**
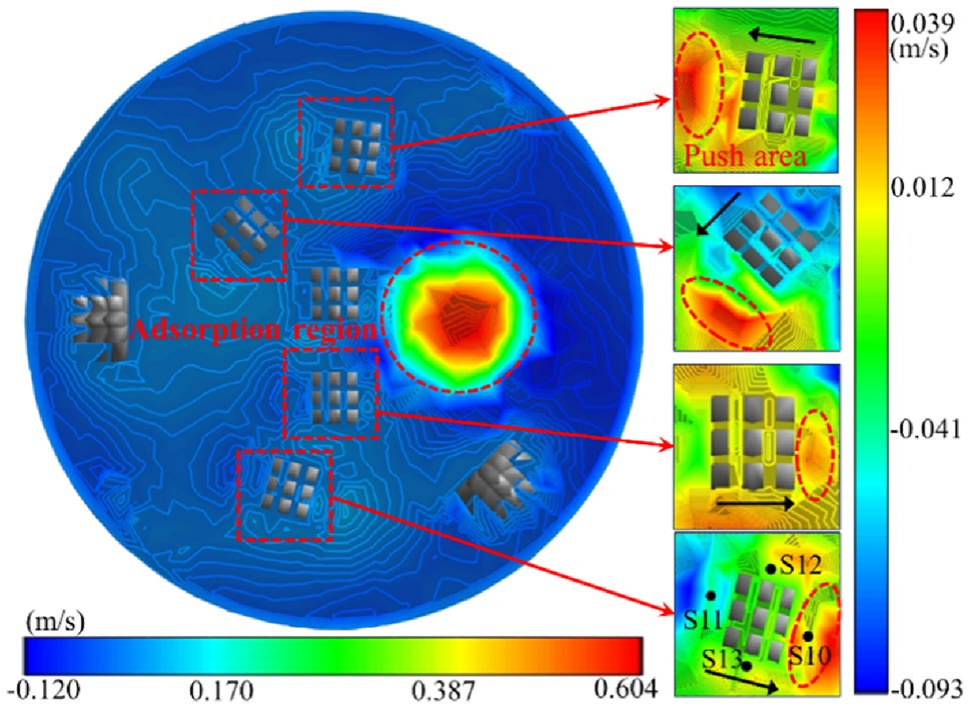
Nephogram of axial velocity of fluid around cutter.

Based on the examination of Fig. 11, when the drill bit was rotated, the cutter can push the mud at the base of the well. The axial velocity of the mud at the head of the cutter experienced a significant increase (Fig. 12), with a greater tangential velocity at the head compared to the tail, and a higher velocity on the outer side relative to the inner side. In addition, the cutter sweeps across the bottom of the well, forming a vortex at its tail, which led to the rapid replenishment of the surrounding mud and a reverse increase in the axial velocity of the mud at the tail. The radial velocity of the mud around the cutter was greater at the tail than at the head, and the inner side experienced a higher velocity than the outer side. The mud flow direction at the head-on and outer sides of the cutter ranged from 19–36 to 22–36 mm from the bottom hole, respectively (Fig. 11b), and the mud runoff deviated from the direction of the slag suction port. Therefore, the movement of the cutter exerted a blocking effect on the radial flow of mud on the head-on and outer sides. At the same clearance depth at the bottom of the well, the mud pressure at the head was lower than that at the tail, and the inside experienced lower pressure than the outside.

### Distribution and migration of rock slag

Based on the numerical simulation results, the distribution state of the rock slag in the bottom hole and the slag discharge pipe at various times was determined, and the migration laws, including convergence, suspension, adsorption, and lifting, were examined.

**Figure 13:**
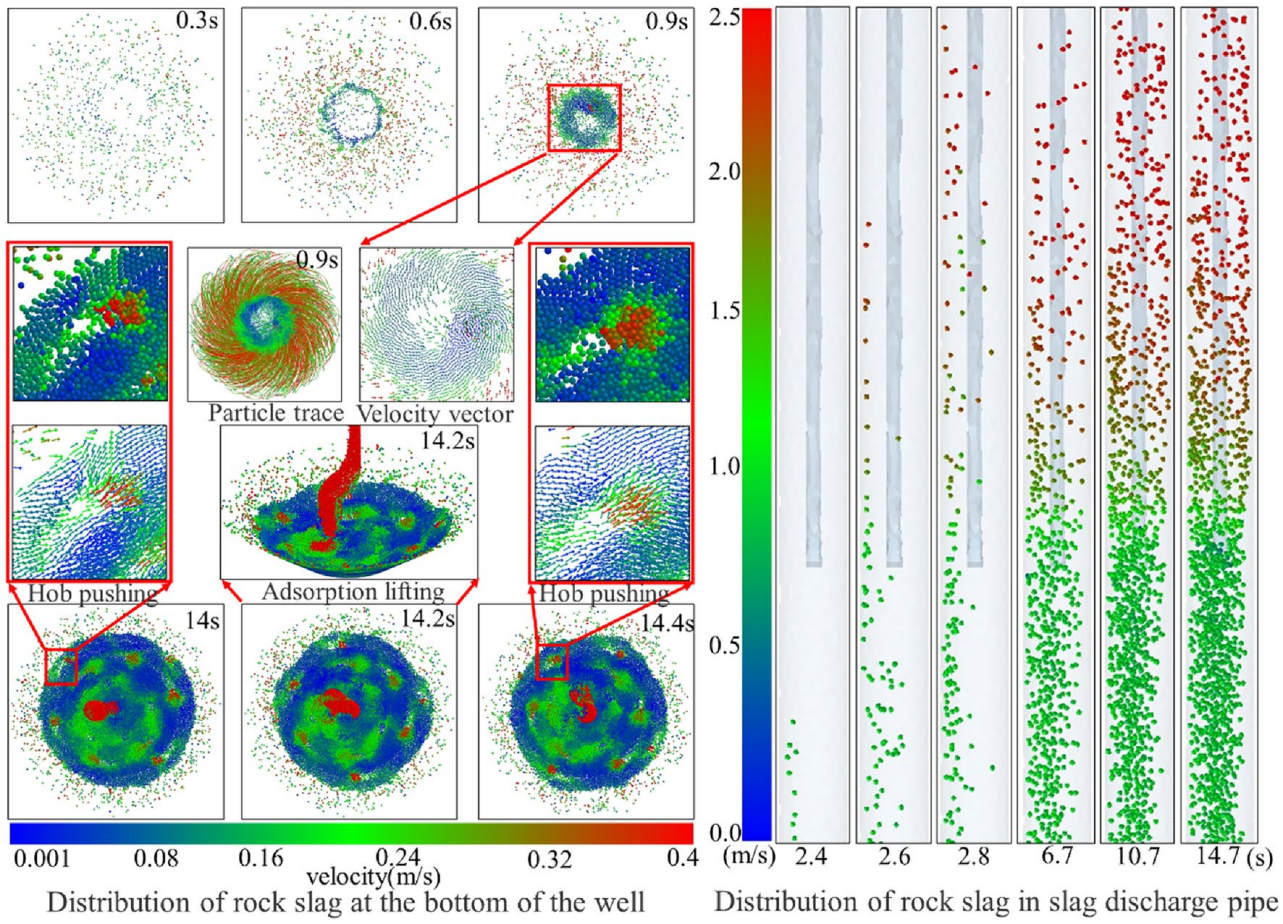
Distribution of rock slag in bottom hole and slag discharge pipe.

From the examination of Fig. 13, the migration of rock slag at the bottom of the well followed a dynamic process comprising of “slip-convergence-suspension-adsorption-lifting”. First, the effects of gravity, bit rotation, and mud scouring caused the broken rock debris to slide rapidly down to the horizontal bottom of the well and accumulate at the interface of the broken rock surface. Subsequently, the push of the cutter and the adsorption of the slag suction port resulted in the rock debris gathering spirally towards the middle of the bottom of the well, overcoming the mud pressure. Finally, the rock slag was pumped out of the bottom hole and entered the slag discharge pipe through the adsorption effect. The cyclic movement of the rock slag at the bottom hole persisted until the clearance of the rock slag was achieved. In the slag discharge pipe, the mud carrying rock flew in the form of “high density and low flow rate” in the liquid–solid section. As it passed through the gas injection end, the manifold became a complex liquid–solid–gas three-phase flow, characterized by a sudden decrease in pressure and three-phase fluid density in the slag discharge pipe. As a result of the combined action of the gas lift and mud column pressure difference, the three-phase fluid was “accelerated by steam injection” and subsequently moved through the slag discharge pipe in the form of “low density and high flow rate”.

### Verification of flow field distribution law

#### Verification of flow field in slag discharge pipe

Based on the similarity theory, the similarity criteria for the model test of gas lift reverse circulation slag removal were derived by dimensional analysis^31^, in which the geometric similarity constant is $C_{{\text{L}}} = 14$, the motion similarity constant is $C_{v} = \sqrt {14}$, the bit rotation speed similarity constant is $C_{\omega } = \sqrt {14} /14$, the gas injection flow volume similarity constant is $C_{Q} = 700$, the mud and rock slag density similarity constant is $C_{\rho } = 1$, the mud pressure similarity constant is $C_{P} = 14$, the mud viscosity similarity constant is $C_{\mu } = 40$, and the time similarity constant is $C_{t} = \sqrt {14}$.

**Figure 14:**
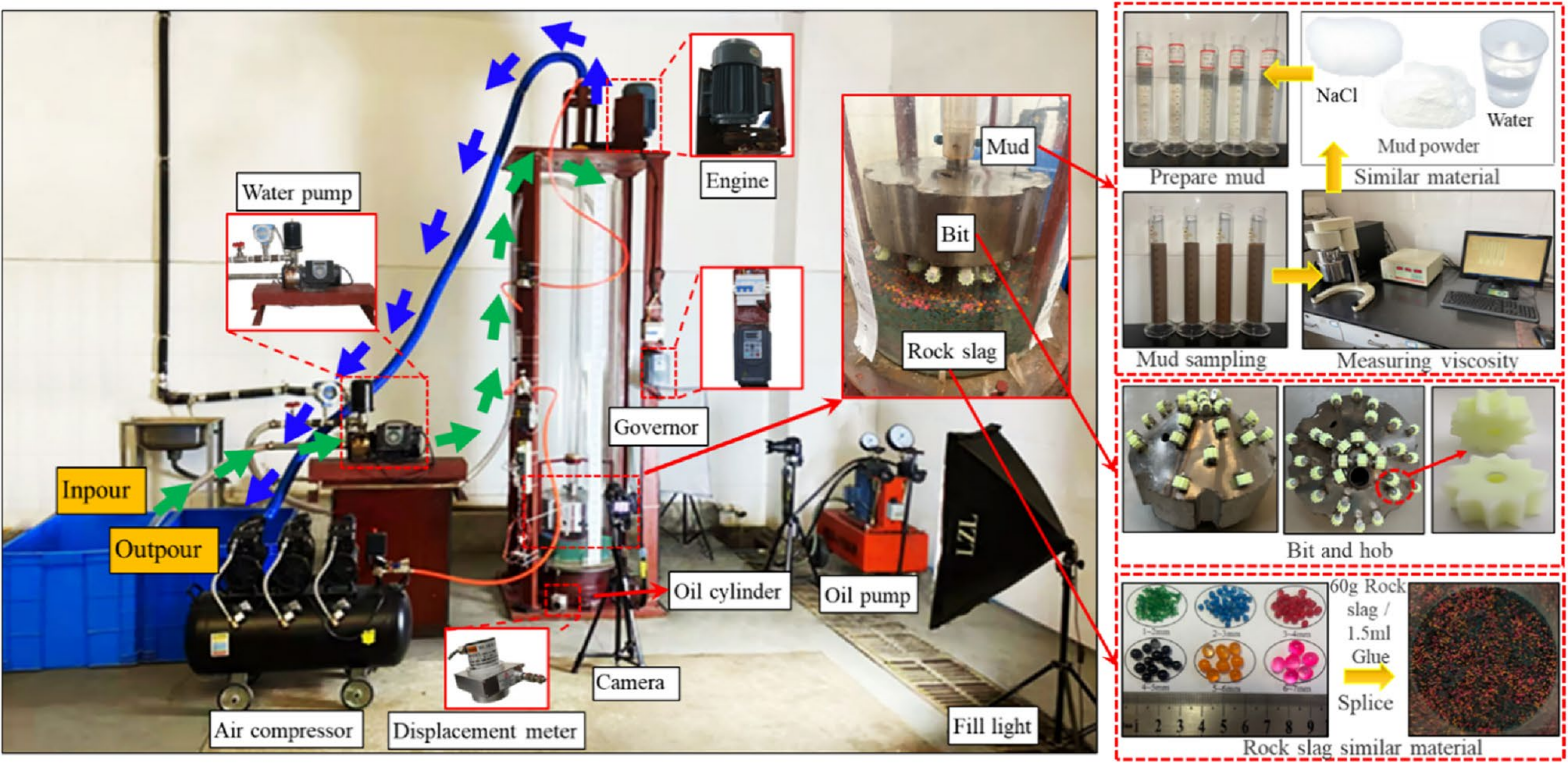
Gas lift reverse circulation slag discharge test device and similar materials.

To investigate the movement law of the multiphase flow field in the gas-lift reverse circulation slag discharge during drilling shaft sinking, a simulation experimental device was developed combined with the construction conditions and criteria of the field drilling method (Fig. 14). The device consisted primarily of a test stand, test chamber, drill bit, drill pipe, air compressor, and an oil pump. The test parameters and operational flow are listed in Table 1 and in Ref.^3^, respectively. Considering the material properties of the mud and rock residue at the Taohutu drilling site, sodium chloride was selected as the weighting agent, mud powder as the tackifier (ratio: 1000 ml water, 0.12 g mud powder, and 154 g sodium chloride), and a “transparent” mud with a density of 1.1 g/cm^3^ and viscosity of 4.4 mPa·s was prepared by mixing it with water. Colored glass balls with a particle size of 1–7 mm were selected and mixed in an equal mass ratio to create a “rock slag-like material with outstanding color, convenient capture, no mud pollution, and reusability”.

**Figure 15:**
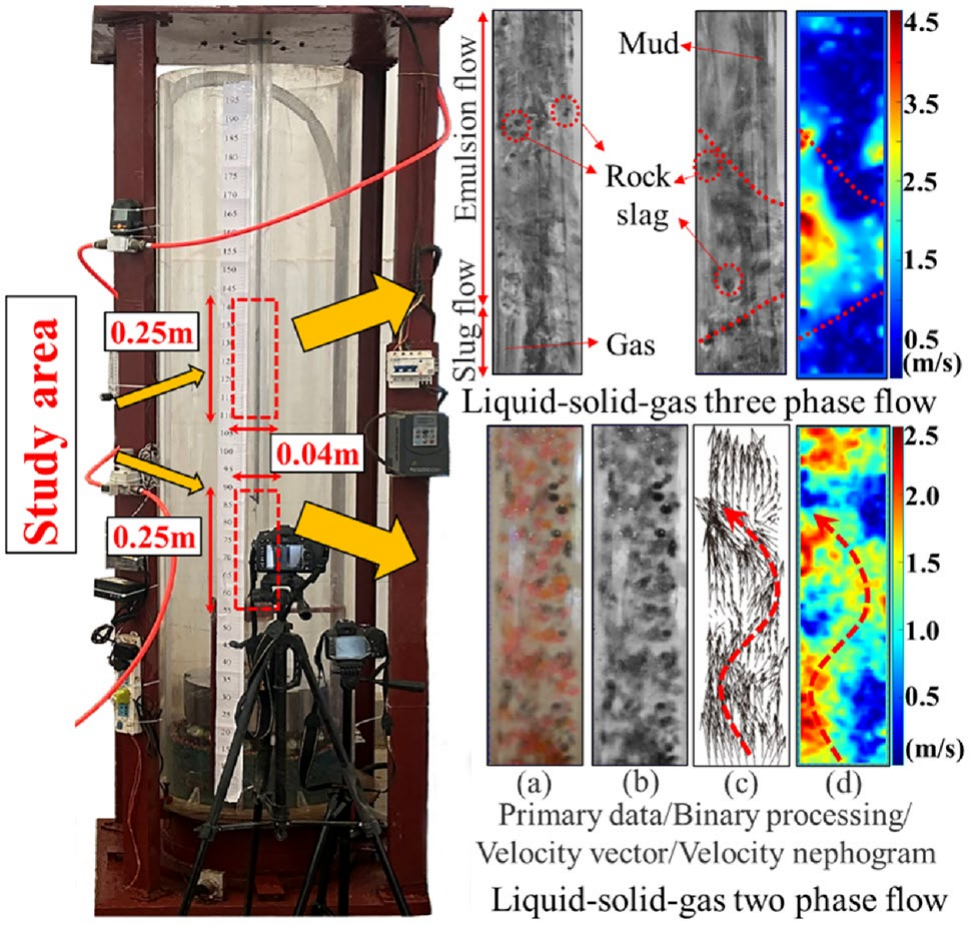
Distribution of multiphase fluid in slag discharge pipe.

**Figure 16:**
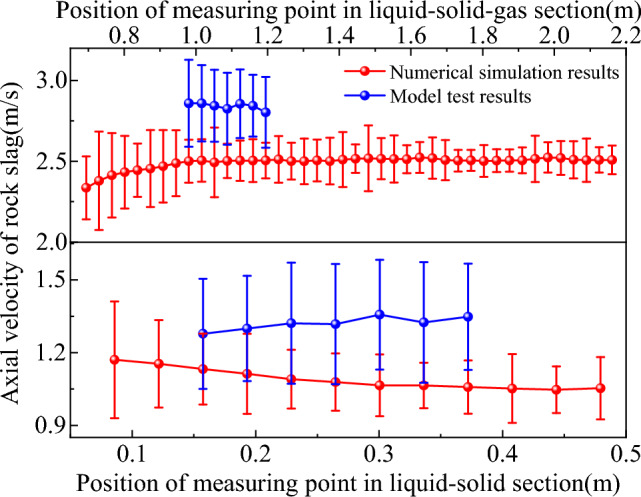
Comparison of axial velocity of rock slag in experimental and simulated flow fields.

Throughout the experiment, a high-frequency camera was employed to monitor and capture the rock slag as it traveled through the slag discharge pipe^32^. The velocity information of the rock slag was obtained using the PIV testing technology^33–35^. Figure 15 illustrates the distribution of the multiphase fluid within the slag discharge pipe, and Fig. 16 presents a comparison of the model test results and numerical simulation for the upward velocity of the rock slag.

From Figs. 15 and 16, mud and rock slag entered the slag discharge pipe from the bottom of the well, ultimately forming a liquid–solid two-phase flow mixed with mud and rock slag. As the two-phase flow passed through the slag discharge pipe and encountered rotation, the wall shear force increased, resulting in a spiral flow pattern, leading to changes in the flow pattern. Upon reaching the gas injection end, a three-phase flow consisting of mud, rock slag, and compressed air was formed, which was primarily characterized by slug and emulsion flows. Emulsion flow occurred because of the bubble rupture within the slug flow. After bubble rupture, the vast majority of the bubbles were irregularly shaped and randomly distributed, contributing to an uneven pressure distribution within the pipe and exacerbating the flow disorder in the liquid–solid–gas section. The model test results indicated that the average upward velocities of rock slag in both the liquid–solid and liquid–solid–gas sections of the slag discharge pipe were 1.32 m/s and 2.85 m/s, respectively. The corresponding numerical simulation results were 1.09 m/s and 2.49 m/s. Compared with the model test results, the relative errors for the upward velocity of rock slag in the liquid–solid and liquid–solid–gas sections of the numerical simulation slag discharge pipe were 17.4% and 12.6%, respectively. The model test results were consistent with the numerical simulation results, thereby validating the accuracy of the flow field distribution law within the slag discharge pipe.

#### Verification of bottom hole flow field

**Figure 17:**
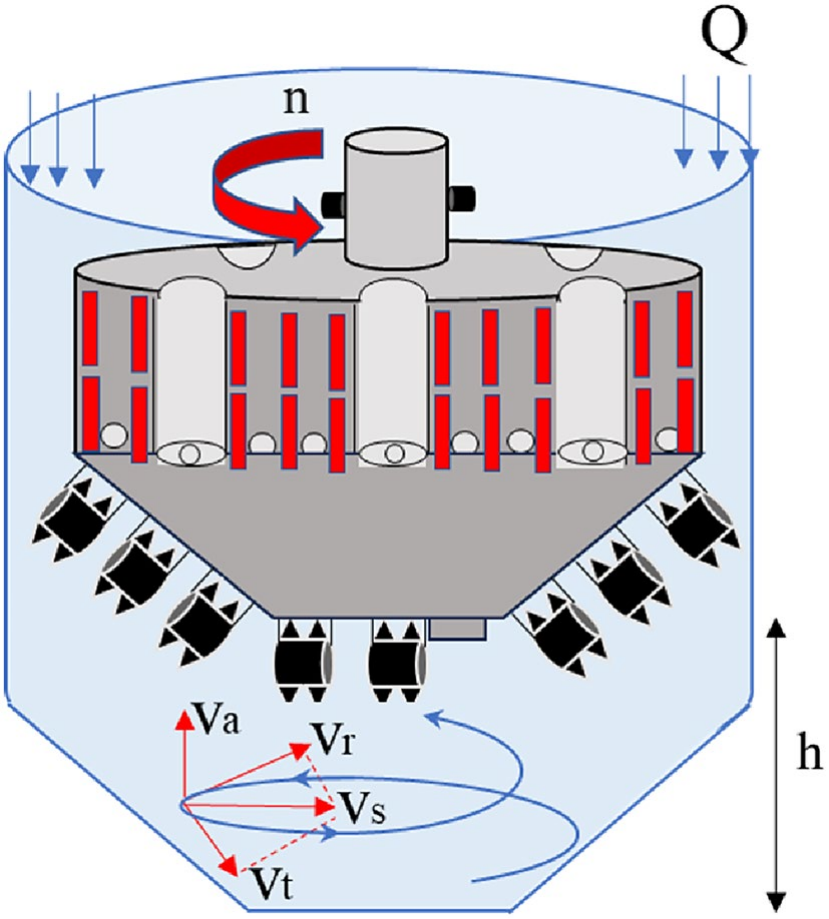
Mathematical model of bottom hole fluid velocity.

**Figure 18:**
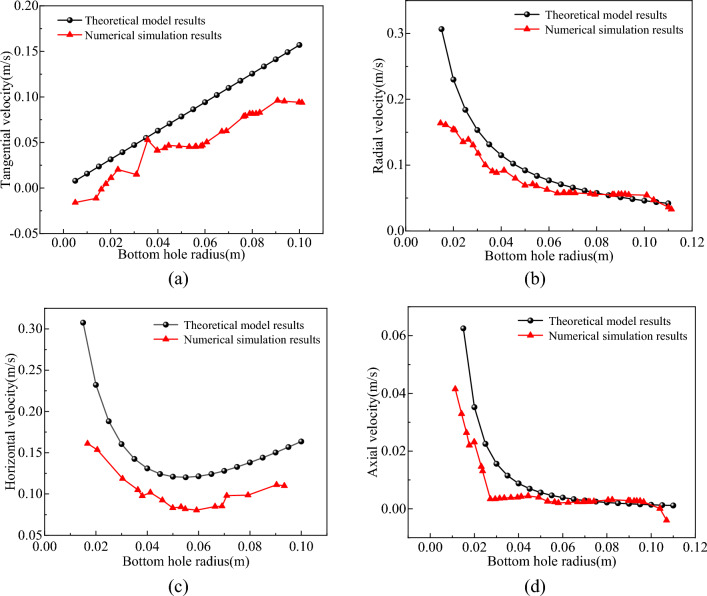
Comparison of bottom hole fluid velocity between numerical simulation and theoretical model. (**a**) Tangential velocity. (**b**) Radial velocity. (**c**) Horizontal velocity. (**d**) Axial velocity.

The velocity of the mud at the bottom of the well includes the tangential velocity $v_{t}$ generated by the rotation of the drill bit and the radial velocity $v_{r}$ flowing to the slag suction port, which are superimposed to form a horizontal velocity $v_{s}$ parallel to the bottom of the well. There is also an axial velocity $v_{h}$ that returns vertically owing to adsorption. Under the combined action of various flows, the circulating mud flow washed the bottom of the well in the form of a spiral flow. As indicated by the flow form and velocity distribution of the mud at the bottom of the well, a velocity model for the bottom-hole fluid was established^36^, as shown in Fig. 17. A comparison between the numerical simulation results and theoretical model results is shown in Fig. 18.

6 $$\left\{ \begin{array}{*{20}l} v_{t} = 2\pi R_{i} nK_{1} /60 \hfill \\ v_{r} = Q/3600(2\pi R_{i} h) \hfill \\ v_{{\text{s}}} = \sqrt {v_{1}^{2} + v_{2}^{2} } \hfill \\ v_{a} = \alpha Q/3600\pi R_{i}^{2} \hfill \\ \end{array} \right.,$$

where $R_{i}$ is any diameter of bottom hole flow field, m; $n$ is the cutter head speed, 30 r/min; $K_{1}$ is an empirical constant, 0.5; $h$ is the distance between the bottom surface of the drill bit and the bottom hole, 0.04 m; $\alpha$ is the velocity distribution coefficient in the vertical direction of the bottom hole, 0.0375; $Q$ is mud circulation flow, 4.24 m^3^/h.

From Fig. 18, the migration of the bottom-hole fluid primarily occurred through horizontal flow, with only vertical upward flow near the slag suction port. The horizontal flow of the bottom hole was predominantly radial, the radial flow of the fluid was most pronounced near the slag suction port, and the tangential velocity of the fluid increased with increasing rotational radius. To confirm the accuracy of the bottom-hole fluid velocity distribution, the standard deviation and relative standard deviation of the numerical simulation results and theoretical model results were analyzed^37^ using the calculation formula shown in Eq. (7) and are shown in Table 2. As shown in Table 2, the relative standard deviation of the bottom hole fluid velocity distribution between the numerical simulation results and theoretical model results ranged within 9.9–25.2%, with an average relative standard deviation of 16.95%. This further confirmed the validity and reliability of the bottom-hole fluid velocity distribution.

7 $$\left\{ \begin{array}{*{20}l} S = \sqrt {\frac{{\sum_{j = 1}^{N} {\left( {v_{k} - v_{l} } \right)^{2} } }}{n - 1}} \hfill \\ f = \frac{S}{{v_{m} }} \hfill \\ \end{array} \right.,$$

where $S$ is the standard deviation; $f$ is the relative standard deviation; $v_{k}$ and $v_{l}$ are the bottom hole fluid velocities of numerical simulation and theoretical model respectively; $N$ is the number of samples; $v_{m}$ is the maximum fluid velocity at the bottom hole.

**Table 2:** Accuracy analysis of bottom hole fluid velocity.

Fluid velocity	$S$/(m/s)	$f$/%
$v_{t}$	0.040	25.2
$v_{r}$	0.046	14.3
$v_{s}$	0.052	18.4
$v_{a}$	0.011	9.9
Average value	0.037	16.95

## Analysis of influencing factors of slag discharge flow field

Drawing upon the velocity and pressure distribution of fluids within the V1 and L1 survey lines situated at the base of the slag discharge pipe as a case study, this study evaluated the impact of various parameters, including the bit rotation speed (20–35 r/min), gas injection volume (6–9 m^3^/h), submerged air duct ratio (0.7–0.84), and mud viscosity (2–8 mPa·s) on the slag discharge flow field distribution in gas-lift reverse circulation.

### Bit rotation speed

**Figure 19:**
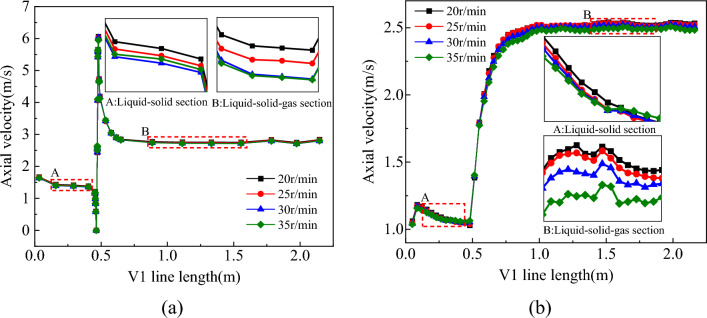
Influence of bit rotation speed on flow field in slag discharge pipe. (**a**) Axial velocity of mud. (**b**) Axial velocity of rock slag.

**Figure 20:**
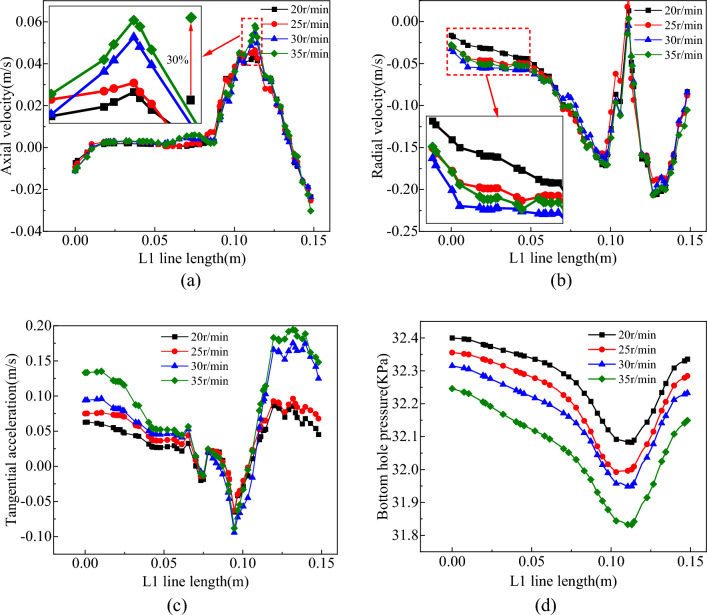
Influence of bit rotation speed on bottom hole flow field. (**a**) Axial velocity on L1 survey line. (**b**) Radial velocity on L1 survey line. (**c**) Tangential velocity on L1 survey line. (**d**) Pressure distribution on L1 survey line.

**Figure 21:**
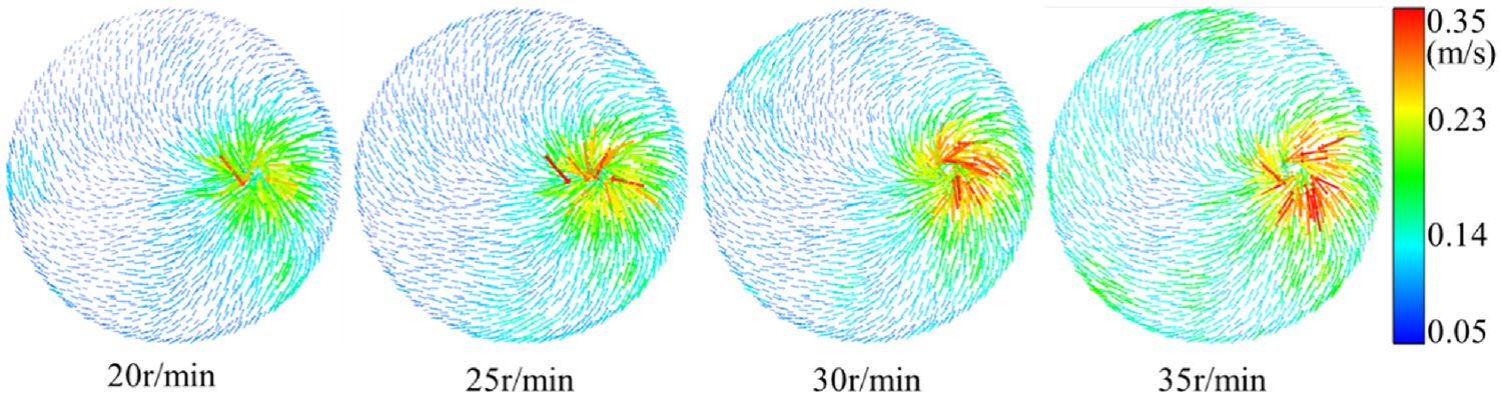
Velocity vector diagram of bottom hole fluid under the influence of different rotating speeds.

**Figure 22:**
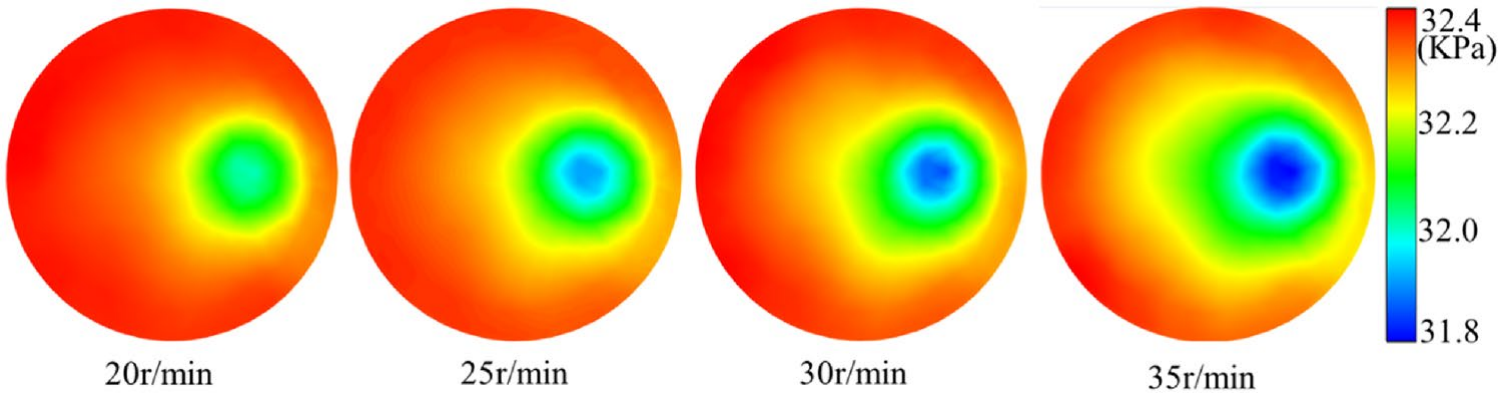
Nephogram of bottom hole fluid pressure distribution under the influence of different rotating speeds.

The distribution of the flow field in the slag discharge pipe and bottom hole when the bit rotation speed was 20–35 r/min is shown in Figs. 19, 20, 21 and 22.

From the examination of Figs. 19, 20, 21 and 22, the axial velocities of the mud and rock slag in the slag discharge pipe were approximately inversely proportional to the bit rotation speed. This was because a high rotational speed intensified the circulation effect of the multiphase flow in the slag discharge pipe and indirectly reduced its axial upward velocity. The rotation speed of the drill bit had the most substantial impact on the velocity and pressure of the bottom-hole fluid. The pressure at the bottom hole slag suction port decreased sharply with increasing bit speed. This results in an augmented pressure difference between the annular mud column and the slag suction port, an enhanced adsorption capacity at the slag suction port, and a substantial increase in the axial velocity of the bottom hole mud. For example, when the rotation speed of the drill bit increases from 20 to 35 r/min, the axial velocity of the mud at the slag suction port increased by 30%. The movement of mud at the bottom of the well was aligned with the rotation of the drill bit, and its tangential velocity increased significantly with increasing rotation speed of the bit. Specifically, when the rotation speed of the bit increased from 20 to 35 r/min, the average tangential velocity of the mud on the L1 survey line increased by 113%. However, the radial velocity of the bottom hole mud initially increased and then decreased with an increase in bit rotation speed. At a rotation speed of 35 r/min, the radial velocity of the mud decreased significantly. This was because an excessive increase in the bit rotation speed enhanced the centrifugal movement of the bottom hole mud, blocked the circulating flow of mud. Hence, a judicious threshold exists for the rotation speed of the drill bit, emphasizing that a greater speed does not necessarily yield superior results^3^.

### Gas injection volume

**Figure 23:**
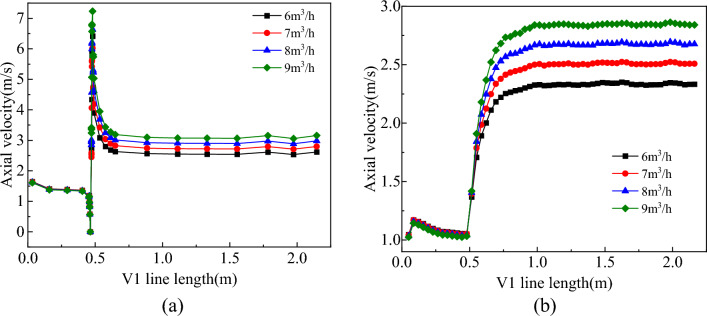
Effect of gas injection volume on flow field in slag discharge pipe. (**a**) Axial velocity of mud. (**b**) Axial velocity of rock slag.

**Figure 24:**
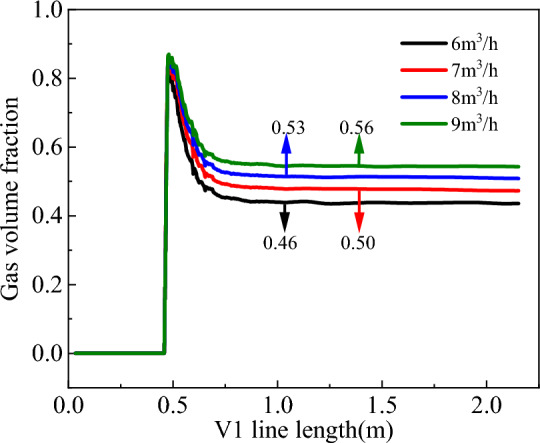
Volume distribution of gas phase on V1 survey line.

**Figure 25:**
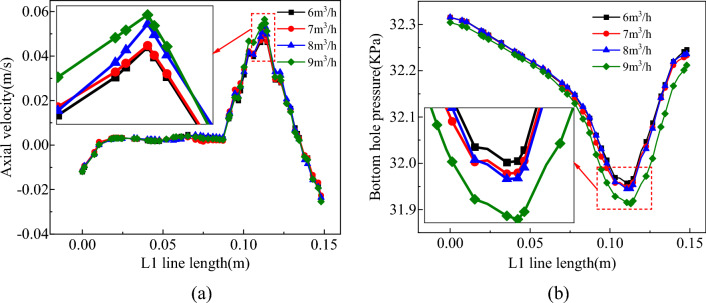
Effect of gas injection volume on bottom hole flow field. (**a**) Axial velocity on L1 survey line. (**b**) Pressure distribution on L1 survey line.

The distribution of the flow field in the slag discharge pipe and bottom hole when the gas injection volume was 6–9 m^3^/h is shown in Figs. 23, 24 and 25.

From Figs. 23, 24 and 25, increasing the gas injection volume significantly enhanced the gas volume fraction in the liquid–solid–gas section of the slag discharge pipe and the axial flow velocity of the mud and rock slag. Specifically, when the gas injection volume increased from 6 to 9 m^3^/h, the gas volume fraction in the slag discharge pipe increased from 0.46 to 0.56, representing a relative increase of 22%. Additionally, the average upward flow velocities of the mud and rock slag in the liquid–solid–gas section increased by 21% and 22%, respectively. This increase can be attributed to the larger kinetic energy of the compressed air, which overcame the gravitational potential energy of the mixed fluid in the slag discharge pipe. The change in the kinetic energy of compressed air into gravitational potential energy and the kinetic energy of the mixed fluid enabled the latter to return at a high flow rate. Moreover, as the gas injection volume increased, the density and pressure of the mixed fluid in the slag discharge pipe rapidly decreased. Under the pressure difference in the external annular slurry column, the mixed fluid in the slag discharge pipe was more likely to achieve a high return velocity. The gas injection volume positively correlated with the axial velocity of the bottom hole fluid and negatively correlated with the bottom hole pressure. When the gas injection volume increased from 6 to 9 m^3^/h, the axial velocity at the bottom hole slag suction port increased by 10.4%, and the pressure difference between the bottom hole and annular mud column increased by 9.3%. Therefore, increasing the gas injection volume was beneficial for enhancing the axial velocity of the bottom hole fluid and the adsorption force of the slag suction port.

### Gas injection volume

**Figure 26:**
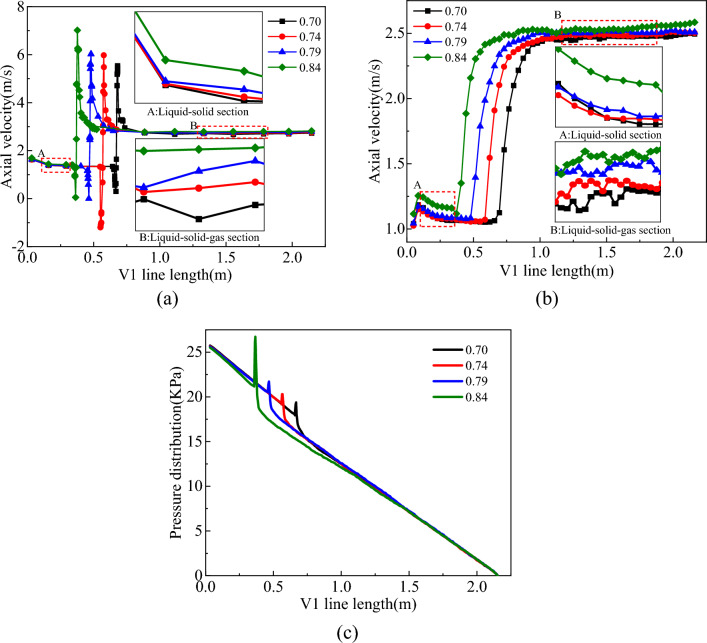
Influence of air duct submergence ratio on flow field in slag discharge pipe. (**a**) Axial velocity of mud. (**b**) Axial velocity of rock slag. (**c**) Pressure distribution on V1 survey line.

**Figure 27:**
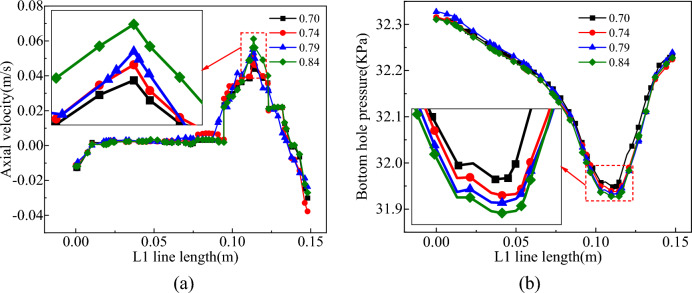
Influence of air duct submergence ratio on bottom hole flow field. (**a**) Axial velocity on L1 survey line. (**b**) Pressure distribution on L1 survey line.

When the air duct submergence ratio was 0.7–0.84, the distribution of the flow field in the slag discharge pipe and the bottom hole is shown in Figs. 26 and 27.

From the analysis of Figs. 26 and 27, increasing the buried depth of the air duct can cause the mud-rock slag two-phase flow to occur “accelerated by gas injection” earlier, and to a certain extent, it can slightly increase the axial velocity of the multiphase fluid in the slag discharge pipe and the bottom hole while reducing the pressure in the slag discharge pipe. When the submergence ratio of the air duct is increased from 0.7 to 0.84, the upward return path of the liquid–solid two-phase flow decreases by 50%, and the upward return path of the liquid–solid gas three-phase flow increased by 15%, increasing the axial velocity of the mud and rock slag. Increasing the submerged length of the air duct can increase the air content below the liquid level in the slag discharge pipe and reduce the pressure in the pipe. Mud flow in the pipe was prone to high flow rates owing to the action of external mud column pressure. On the other hand, the rock slag-mud two-phase flow was “accelerated by gas injection” earlier and was discharged to the ground at a high flow rate, which effectively reduced the return time of rock slag and mud in the slag discharge pipe and enhanced the slag discharge efficiency.

### Mud viscosity

**Figure 28:**
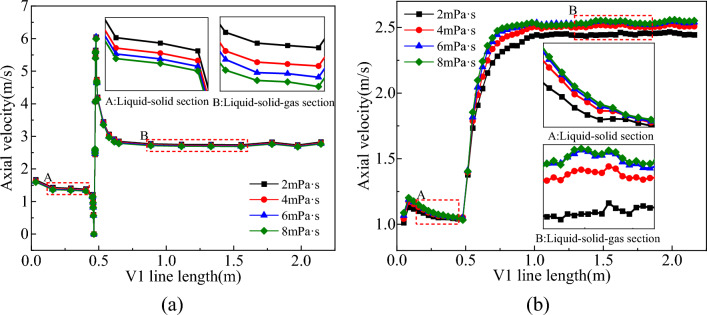
Effect of mud viscosity on flow field in slag discharge pipe. (**a**) Axial velocity of mud. (**b**) Axial velocity of rock slag.

**Figure 29:**
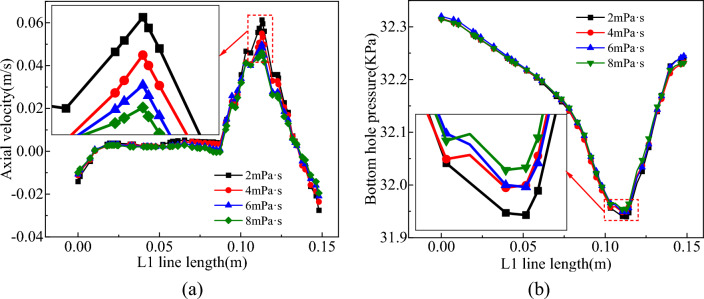
Effect of mud viscosity on bottom hole flow field. (**a**) Axial velocity on L1 survey line. (**b**) Pressure distribution on L1 survey line.

With the mud viscosity ranging in 2–8 mPa·s, the distribution of the flow field in the slag discharge pipe and bottom hole is shown in Figs. 28 and 29.

Based on the analysis of Figs. 28 and 29, the axial velocity of mud in the slag discharge pipe and at the bottom hole was negatively correlated with the mud viscosity, while the axial velocity of rock slag was directly proportional to the mud viscosity. Low-viscosity mud was capable of achieving higher velocities and being lifted, resulting in a low pressure in the slag discharge pipe and bottom hole. However, its rock-carrying capacity was limited. For instance, when the mud viscosity was 2 mPa·s, the axial velocity of the mud at the slag suction port increased by 25% compared with that at a viscosity of 8 mPa·s. Nevertheless, the average axial velocity of rock slag in the slag discharge pipe decreased by 5%. This occurred because the wall shear force between the low-viscosity mud and slag discharge pipe, drill bit, and well wall was minimal, favoring mud circulation. Additionally, the low-density mixed fluid in the liquid–solid–gas section of the slag discharge pipe maintained a low-pressure state. Nonetheless, owing to its low viscosity, low wall shear force with rock slag, low axial velocity of rock slag, and poor rock-carrying capacity, mud viscosity cannot be reduced to the minimum level. In actual drilling engineering, it was essential to consider the ability of mud to balance ground pressure, protect the well wall, suspend the shaft wall, and carry rock slag.

## Conclusion

To investigate the distribution law of the liquid–solid–gas multiphase coupling slag discharge flow field in the gas-lift reverse circulation slag discharge, a numerical model of the gas-lift reverse circulation slag discharge was established using the CFD–DEM research method. This model revealed the distribution law of the flow field in the bottom hole and slag discharge pipe during drilling, and was validated through model testing and theoretical modeling. Finally, the factors affecting the slag discharge flow field were analyzed by altering the drilling construction parameters. Based on this study, the following conclusions were drawn:

1. When drilling the north wind shaft in Taohutu, the mud at the bottom of the well primarily moved horizontally in the horizontal plane and radially in the inclined plane. The axial upward flow of mud was concentrated in the adsorption area of the slag suction port. The radial flow of mud on both sides of the slag suction port was the most significant. The movement of the three-phase mixed fluid in the slag discharge pipe was primarily axial. When passing through the gas injection end, the pressure and density of the mixed fluid suddenly decreased, and the fluid velocity increased sharply. The dynamic migration of rock slag at the bottom of the well was characterized by “slip-convergence-suspension-adsorption-lifting”. The liquid–solid section and liquid–solid–gas section of the slag discharge pipe flew in the states of “high density, low flow rate” and “low density, high flow rate”, respectively.
2. Utilizing the experimental device constructed based on the self-devised drilling method for well drilling gas lift reverse circulation slag discharge, as well as the established bottomhole fluid velocity distribution model, the distribution of the multiphase coupled slag discharge flow field was validated. The results indicated that the relative errors for the upward velocity of the rock slag in the liquid–solid and liquid–solid gas sections of the slag discharge pipe were 17.4% and 12.6%, respectively. The relative standard deviation of the bottomhole fluid velocity distribution was between 9.9 and 25.2%, with an average relative standard deviation of 16.95%.
3. The rotation speed of the drill bit exerted the most significant impact on the velocity and pressure of the bottom hole fluid. Increasing the rotation speed of the drill bit can significantly increase the tangential velocity of the bottom hole fluid and the pressure difference between the bottom hole and the annular mud column. However, excessive rotation speeds can block mud circulation and reduce runoff velocity. Increasing the gas injection rate can significantly increase the gas volume fraction in the liquid–solid–gas section of the slag discharge pipe and the axial velocity of the multiphase fluid. Furthermore, increasing the buried depth of the air duct can accelerate the two-phase flow of rock slag and mud be “accelerated by gas injection” in advance, effectively reducing the return time of rock slag and mud in the slag discharge pipe and improving the slag discharge efficiency. Notably, low-viscosity mud can easily achieve a high return velocity, but its rock carrying capacity can be poor.

## Data Availability

Data is provided within the manuscript.

## References

[1] Yuan L (2023). Strategic conception of carbon neutralization in coal industry. Strateg. Study CAE.

[2] Wang GF, Pang YH, Xu YX, Meng LY, Han HJ (2023). Development of intelligent green and efficient mining technology and equipment for thick coal seam. J. Min. Saf. Eng..

[3] Cheng H, Guo LH, Yao ZS, Wang ZJ, Rong CX (2023). Experimental study on transport law of multiphase slag discharge and optimization of well washing parameters in gas lift reverse circulation of drilling shaft sinking. J. China Univ. Min. Technol..

[4] Fan JD, Feng H, Song CY, Ren HW, Ma Y, Wang QC, Tan J, Liu QH, Li C (2022). Key technology and equipment of intelligent mine construction of whole mine mechanical rock breaking in Kekegai Coal mine. J. China Coal Soc..

[5] Liu YW, Lin GJ, Du BB, Guan ZC, Zhao GS (2023). Ground measurement of noise characteristics in drill string during drilling. J. Petrol. Sci. Eng..

[6] Lin YB, Qin Y, Ma DM, Wang SQ, Qiao JW (2023). In situ stress variation and coal reservoir permeability response of the Jurassic Yan? An formation in the southwestern Ordos basin, China: Its impact on coalbed methane development. J. Petrol. Sci. Eng..

[7] Liu ZQ, Song CY, Ji HG, Liu SJ, Tan J, Cheng SY, Ning FB (2021). Construction mode and key technology of mining shaft engineering for deep mineral resources. Int. J. Min. Sci. Technol..

[8] Lashgari A, Fouladgar MM, Yazdani-Chamzini A, Skibniewski MJ (2011). Using an integrated model for shaft sinking method selection. J. Civ. Eng. Manag..

[9] Nwanwe OI, Teodoriu C (2020). Matrix selection and comparison for selecting drilling methods and technologies for a wide range of applications. J. Petrol. Sci. Eng..

[10] Cui MY, Liu JY, Li XY, Huang JY (2022). Analysis on mine shaft drilling method applied to mine shaft sinking in Western China area—Taking the construction of air inlet shaft in Kekegai coal mine as an example. Min. Constr. Technol..

[11] Li AY, Zhang K, Xu JF, Xie XY, Kang YF (2023). Stability of surrounding rock and support optimization of a broken soft-rock-inclined shaft bored by tunnel boring machine: A case study of Kekegai sub-inclined shaft. Tunn. Constr..

[12] Liu SW, Feng YL, Dong SJ (2013). Influence factors analysis and parameters optimization of deslagging in roadway floor anchoring boreholes. J. China Univ. Min. Technol..

[13] Feng YL, Ju WJ, Jiao JK, Zhang SW (2017). Study on pump suction reverse circulation deslagging in drilling roadway floor anchoring boreholes through numerical experiments. J. Min. Saf. Eng..

[14] Zhang H, Li GS, Jiang SQ (2020). Study of the mechanism of strong slag discharge in deep hole drilling of small-bore anchoring hole in roadway floor. J. Exp. Mech..

[15] Fu MX, Liu SW, Jia HX, Jiang YJ, Lu YH (2022). Study on migration rules and influencing factors of the fragments ascending during boreholes drilling rock in coal mine roadway floors. J. Min. Saf. Eng..

[16] Huang Y, Yin K, Zhu LH, Bo K (2011). Velocity and pressure model of large-diameter DTH hammer pneumatic reverse circulation fluid. J. Jilin Univ. (Earth Sci. Ed.).

[17] Huang ZQ, Zhou Y, Shan DW, Yang MJ, Liu SB, Bu Y (2008). Study on influence of layout of DHT bits junk slot on hole-bottom flow field. Eq. Geophys. Prospect..

[18] Qiu, H. B. *Numerical Simulation of the Bottom Flow Field in the Gas-Circulation Media*. Ph.D. thesis, China University of Petroleum (2013).

[19] Yao, J. L. *Investigations of Flow Field in Annulus and Erosion Characteristics on the Pipe with Cuttings in Gas Drilling*. Ph.D. thesis, Shanghai University (2011).

[20] Jiao N, Wang YS, Meng CX (2020). Numerical simulation on the flow field and slag carrying efficiency of air flush drilling for vertical shaft boring machine. J. China Coal Soc..

[21] Chen, Z. L. *Study on Flow Field and Rock Carrying Efficiency of Foam Washing in Vertical Shaft Boring Machine*. Ph.D. thesis, China University of Mining and Technology (2020).

[22] Meng, C. X. *Study on Liquid Cleaning System and Flow Field of Vertical Shaft Boring Machine*. Ph.D. thesis, China University of Mining and Technology (2019).

[23] Krause B, Liedmann B, Wiese J, Wirtz S, Scherer V (2015). Coupled three dimensional DEM-CFD simulation of a lime shaft kiln-Calcination, particle movement and gas phase flow field. Chem. Eng. Sci..

[24] Pang BX, Wang SY, Lu CL, Cai WJ, Jiang XX, Lu HL (2019). Investigation of cuttings transport in directional and horizontal drilling wellbores injected with pulsed drilling fluid using CFD approach. Tunn. Undergr. Space Tech..

[25] Abdulkadir M, Hernandez-Perez V, Lo S, Lowndes IS (2015). Comparison of experimental and computational fluid dynamics (CFD) studies of slug flow in a vertical riser. Exp. Therm. Fluid Sci..

[26] Wang H, Hao SJ, Mo HT (2017). The pilot study on start pressure of the air compressor during the air-lift reverse circulation drilling. Coal Geol. Explor..

[27] Huang Y, Yin K, Zhu LH (2012). Mathematical model for cuttings migration in center channel of DTH hammer of reverse circulation drilling. Coal Geol. Explor..

[28] Lee M, Park G, Park C, Kim C (2020). Improvement of grid independence test for computational fluid dynamics model of building based on grid resolution. Adv. Civ. Eng..

[29] Liu ZQ, Song CY, Cheng SY, Jing GY, Zhao JX, Chen Y, Zhao LK (2023). Research on the technology and equipment system of large diameter shaft drilling based on gravity slagging. Coal Sci. Technol..

[30] Xia M, He JJ, Wang ZJ, Song HH (2013). Numerical simulation on flow field of shaft bottom drilled with large-diametered drill bit. Min. Process Eq..

[31] Protsenko VS, Danilov FI (2012). Application of dimensional analysis and similarity theory for simulation of electrode kinetics described by the Marcus–Hush–Chidsey formalism. J. Electroanal. Chem..

[32] López J, Pineda H, Bello D, Ratkovich N (2016). Study of liquid–gas two-phase flow in horizontal pipes using high speed filming and computational fluid dynamics. Exp. Therm. Fluid Sci..

[33] Pérez JM, Sastre F, Clainche S, Velázquez A, Vega JM (2022). Three-dimensional flow field reconstruction in the wake of a confined square cylinder using planar PIV data. Exp. Therm. Fluid Sci..

[34] Simonini A, Theunissen R, Masullo A, Vetrano MR (2019). PIV adaptive interrogation and sampling with image projection applied to water sloshing. Exp. Therm. Fluid Sci..

[35] Ergin FG (2021). An automatic static masking technique using particle image velocimetry image ensembles. Exp. Therm. Fluid Sci..

[36] Zhang YC, Sun J, Wang AS (2008). Drilling Technology.

[37] Wang BL, Xu JL, Xuan DY (2018). Time function model of dynamic surface subsidence assessment of grout-injected overburden of a coal mine. Int. J. Rock Mech. Min. Sci..

